# Core Amino Acid Residues in the Morphology-Regulating Protein, Mms6, for Intracellular Magnetite Biomineralization

**DOI:** 10.1038/srep35670

**Published:** 2016-10-19

**Authors:** Ayana Yamagishi, Kaori Narumiya, Masayoshi Tanaka, Tadashi Matsunaga, Atsushi Arakaki

**Affiliations:** 1 Division of Biotechnology and Life Science, Institute of Engineering, Tokyo University of Agriculture and Technology, Koganei, Tokyo 184-8588, Japan; 2 Department of Chemical Science and Engineering, School of Materials and Chemical Technology, Tokyo Institute of Technology, Meguro-ku, Tokyo 152-8550, Japan

## Abstract

Living organisms produce finely tuned biomineral architectures with the aid of biomineral-associated proteins. The functional amino acid residues in these proteins have been previously identified using *in vitro* and *in silico* experimentation in different biomineralization systems. However, the investigation in living organisms is limited owing to the difficulty in establishing appropriate genetic techniques. Mms6 protein, isolated from the surface of magnetite crystals synthesized in magnetotactic bacteria, was shown to play a key role in the regulation of crystal morphology. In this study, we have demonstrated a defect in the specific region or substituted acidic amino acid residues in the Mms6 protein for observing their effect on magnetite biomineralization *in vivo*. Analysis of the gene deletion mutants and transformants of *Magnetospirillum magneticum* AMB-1 expressing partially truncated Mms6 protein revealed that deletions in the N-terminal or C-terminal regions disrupted proper protein localization to the magnetite surface, resulting in a change in the crystal morphology. Moreover, single amino acid substitutions at Asp123, Glu124, or Glu125 in the C-terminal region of Mms6 clearly indicated that these amino acid residues had a direct impact on magnetite crystal morphology. Thus, these consecutive acidic amino acid residues were found to be core residues regulating magnetite crystal morphology.

Biomineralization is an elaborate process that controls the size, shape, surface, and composition of inorganic structures during their synthesis under ambient conditions^1^. Because these structural factors strongly influence the chemical and physical properties of materials^2^,^3^, biomineralization has attracted considerable attention in the field of materials science, as well as that of basic sciences^4^,^5^,^6^. Proteins are known to play a major role in the key processes of biomineralization, such as nucleation^7^, regulation of the shape^8^, and assembly of crystals^9^. Some of these proteins are considered to be specifically associated with biominerals and regulate their morphology during their formation. Recently, structural investigations on biomineral-associated proteins by X-ray structural analysis^10^, solid-state nuclear magnetic resonance (NMR)^11^, and molecular dynamic simulation^8^ have helped to develop models depicting biomineral-protein interactions. These models indicate that the acidic amino acid residues (or regions containing these residues) that are a common characteristic in several proteins are responsible for the interaction with the biomineral crystal surface^6^,^8^,^10^,^12^. Thus, towards the further elucidation of biomineral morphology regulation, the detailed investigation of acidic amino acids in biomineral-associated proteins in living organisms is required.

Magnetotactic bacteria synthesize magnetite crystals with species-specific sizes and morphologies, such as cubo-octahedra, elongated hexahedra, and bullet shapes, under various environmental conditions^13^,^14^. This has given rise to a theory that magnetotactic bacteria significantly regulate the magnetite biomineralization process using specifically produced biological molecules. The magnetite crystals are synthesized in the subcellular organelle (magnetosome) and enveloped by the magnetosome membrane containing its specific proteins. Genome analysis^15^,^16^,^17^,^18^ and proteome analysis of magnetosome membrane proteins^19^,^20^,^21^,^22^ revealed the key molecules responsible for the magnetosome formation. In addition, various genetic techniques, including transformation and recombination, have been established for this organism over the past two decades^23^,^24^,^25^,^26^. Therefore, magnetotactic bacteria have become one of the ideal model organisms for the *in vivo* study of the biomineralization mechanism using various molecular techniques^25^,^26^,^27^,^28^.

In our previous study, we identified a series of proteins: Mms5, Mms6, Mms7, and Mms13, localized onto magnetite crystals in the *Magnetospirillum magneticum* strain AMB-1^20^. Their amino acid sequences contain a C-terminal hydrophilic region comprising of acidic amino acids, and an N-terminal hydrophobic region, with a Gly and Leu (GL) repetitive region. A functional analysis of Mms6 in living cells, conducted by establishing a *mms6* gene deletion mutant, revealed that the gene deletion mutant produced elongated and smaller magnetite crystals than the wild-type cells^29^,^30^. Involvement of Mms6 and other Mms proteins in the regulation of crystal morphology of magnetite was elucidated^30^. In contrast, *in vitro* chemical synthesis of magnetite crystals using Mms6, revealed the formation of particulate crystals (cubo-octahedron), similar to those formed in *Magnetospirillum* spp., whereas rectangular crystals (octahedron) were obtained in the absence of this protein^31^,^32^. Magnetite synthesis using synthetic short peptides mimicking Mms6 suggested that the acidic amino acids influence the function of Mms6 in regulating crystal morphology^32^,^33^. Iron binding^20^,^34^,^35^ and iron oxide nucleation at the C-terminal acidic region were also confirmed^36^. According to these *in vitro* studies, the acidic amino acids in the C-terminal region are most likely to be responsible for controlling the crystal morphology. However, the key residue responsible for the function of Mms6 remains unclear. In addition, the function of the acidic residues in the living organism has not yet been elucidated.

In this study, we established and analyzed a series of gene deletion mutants and transformants of *M. magneticum* strain AMB-1, expressing partially truncated or largely deleted Mms6 proteins, using two different strategies. Moreover, a single amino acid substitution in the C-terminal region of Mms6 was investigated in order to identify the amino acid residues essential for the function of Mms6.

## Results

### Morphological characterization of magnetite crystals formed in the partial *mms6* gene deletion mutants

In our previous study, the *mms6* gene deletion mutant strain (Δ*mms6* strain) was found to synthesize elongated magnetite crystals with a smaller size and lower shape factor than that of the cubo-octahedral crystals synthesized by the wild-type strain^29^. This indicated that Mms6 plays a role in the *in vivo* regulation of crystal morphogenesis (imparting the cubo-octahedral shape). In this study, two approaches were adopted to express the partially deleted proteins in the bacterial cell in order to identify the functional region of this protein. The gene encoding the partially deleted Mms6 protein was replaced with the *mms6* gene in the AMB-1 genome by homologous recombination. Alternatively, a plasmid vector harboring the partially deleted *mms6* gene was complemented into the Δ*mms6* strain.

**Figure 1:**
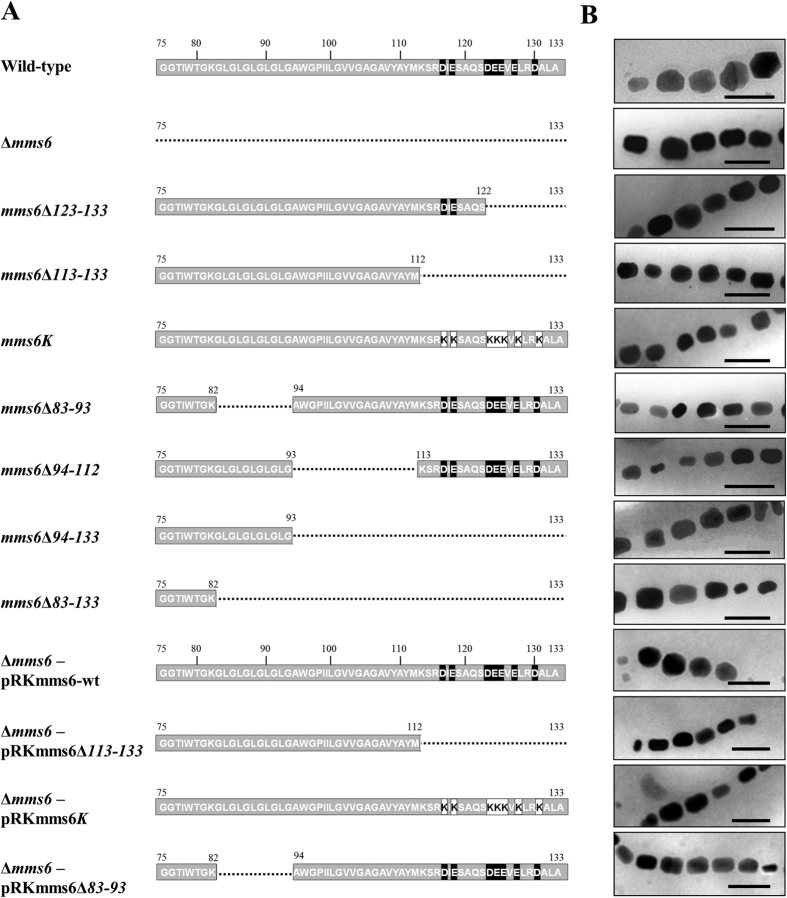
Morphology of magnetite crystals expressed in recombinants carrying partially deleted or point mutated Mms6 proteins developed by homologous recombination or complementation of partially deleted Mms6 protein. (**A**) Overview of partial deletion or mutation constructs. The numbers indicate the number of the amino acid residue. The black and white boxes denote the acidic amino acid and lysine residues, respectively. The dashed lines represent the deleted region in Mms6 variants. (**B**) Transmission electron micrographs of magnetite crystals from wild-type, *mms6*Δ*123–133, mms6*Δ*113–133, mms6K, mms6*Δ*83–93, mms6*Δ*94–112, mms6*Δ*94–133, mms6*Δ*83–133*, Δ*mms6*, Δ*mms6* carrying pRKmms6-wt, pRKmms6Δ113*–*133, pRKmms6K, and pRKmms6Δ83*–*93 strains. Scale bar, 100 nm.

Mms6 variants containing a truncated C-terminal region (*mms6*Δ*123–133, mms6*Δ*113–133, mms6*Δ*94–133*, or *mms6*Δ*83–133*) or an internal deletion (*mms6*Δ*83–93* or *mms6*Δ*94–112*) consisting of a putative transmembrane region were established by homologous recombination (Fig. 1A). Transmission electron microscopy (TEM) revealed the presence of aligned chains of magnetite crystals in all the mutant and wild-type strains (Fig. 1B). The mutant strains carrying a C-terminally truncated Mms6 (*mms6*Δ*123–133* and *mms6*Δ*113–133* strains) formed rod-shaped magnetite crystals, similar to that seen in the Δ*mms6* strain (Fig. 1B). The average diameters and shape factors of crystals formed by the *mms6*Δ*113–133* and *mms6*Δ*123–133* strains were 30.7 ± 9.4 and 31.8 ± 15.2 nm and 0.76 ± 0.14 and 0.76 ± 0.12, respectively (Table 1). The *mms6*Δ*113–133* and *mms6*Δ*123–133* strains showed no significant differences (Mann-Whitney *p*-value > 0.05) (Supplementary Table S4); therefore, the morphological change in the crystals was attributed to the truncation of amino acid residues 123–133. The mutant strain carrying an Mms6 protein wherein 7 acidic amino acids in the C-terminal region were substituted with lysine residues (*mms6K* strain) also synthesized rod-shaped crystals (Fig. 1B). This coincided with the crystals formed by the *mms6*Δ*113–133* and *mms6*Δ*123–133* strains (Table 1). These results suggested that the acidic amino acids in the C-terminal region form the functional domain of Mms6. Similarly, the mutant strains carrying an internal deletion (*mms6*Δ*83–93* and *mms6*Δ*94–112* strains) or a large deletion in the C-terminal region (the *mms6*Δ*94–133* and *mms6*Δ*83–133* strains) produced rod-shaped crystals (Fig. 1B). However, their average shape factor (0.63–0.66) was slightly smaller than that observed in the *mms6*Δ*83–93* strain and C-terminal deletion mutant strains (Mann-Whitney p-value < 0.05) (Table 1 and Supplementary Table S4).

We used SHAPE program^36^ (http://lbm.ab.a.u-tokyo.ac.jp/~iwata/shape/) for quantitative evaluation of shape, based on elliptic Fourier descriptors (EFD). By using this program, the contours of objects are extracted by EFD, and then the obtained coefficients of EFD are normalized and summarized by principal component analysis. The obtained principal component scores (PC1 and PC2) were used to compare the crystals in the wild-type, *mms6*Δ*123–133, mms6*Δ*94–133*, and *mms6*Δ*83–93* strain. The first principal component (PC1) and PC2 accounted for 66.1% and 6.2% of the total variation in crystal shape, respectively (Supplementary Figure 2). PC1 represented crystal length/width ratio; crystals with a low PC1 score were elliptical, whereas crystals with a high PC1 score were orbicular. The PCA plot of PC1 vs PC2 indicated that the crystal shape changed from orbicular to elliptical with the deletion of the amino acid residues 123*–*133. Additionally, the plot for *mms6*Δ*83–93*, and *mms6*Δ*94–133* showed that the crystals in these strains were elliptical.

The plasmid harboring a partially deleted *mms6* gene was transformed into the Δ*mms6* strain and complementation of the gene function in the cells was evaluated as an alternative approach towards identifying the functional domain in the Mms6 protein sequence. The transformant strain harboring the pRKmms6-wt plasmid that encoded the full-length Mms6 protein synthesized spherical crystals (Fig. 1B). The crystal size and shape factor of the Δ*mms6* strain (harboring pRKmms6-wt) were 41.4 ± 14.0 nm and 0.88 ± 0.09 (Table 1), respectively, indicating successful complementation of the partially deleted *mms6* gene function. In contrast, the genes encoding Mms6 proteins with partial deletions in the N-terminal GL region (pRKmms6Δ83*–*93) or C-terminal hydrophilic region (pRKmms6Δ113*–*133), or with acidic amino acids in the C-terminal substituted with lysine residues (pRKmms6K) synthesized rod-shaped crystals (Fig. 1B). The average size of the crystals produced by these transformants was approximately 75–78% that of the crystals produced by the Δ*mms6* strain harboring pRKmms6-wt. The shape factors of crystals produced by these 3 strains (approximately 0.66) were apparently distinct from those of the wild-type strain (0.88 ± 0.10) and Δ*mms6* (harboring pRKmms6-wt; 0.88 ± 0.09) (Mann-Whitney *p*-value < 0.05) (Supplementary Table S4). These results are consistent with the observations in the partial gene deletion mutants obtained by homologous recombination (Table 1).

All the partial deletion strains (established by homologous recombination and transformation), such as the Δ*mms6* strain and other *mms* gene deletion mutants, developed in this study produced elongated rod-shaped crystals^29^,^30^. The average major and minor axes were found to be slightly variable among mutant strains established by homologous recombination. These differences could be attributed to the differences in their growth conditions or the effect of the partial deletion. However, the 7 partial deletion strains could be separated into 2 groups based on the average shape factor. The shape factor of the first group, comprising of proteins with deletions in the C-terminal or GL region (*mms6*Δ*113–133, mms6*Δ*123–133, mms6K*, and *mms6*Δ*83–93* strains), was in the range 0.70–0.76. The shape factor of the second group, containing proteins with large deletions (*mms6*Δ*94–112, mms6*Δ*94–133*, and *mms6*Δ*83–133* strains), ranged from 0.63 to 0.66, which was similar to that of the Δ*mms6* strain. Therefore, the large deletions in the latter group resulted in the complete elimination of the function of Mms6 protein; in contrast, the Mms6 protein continued to function despite deletions in the domains, or acidic or GL regions, owing to the rest of the region. Deficiencies in the C-terminal and GL repetitive regions affected the magnetite crystal morphology, indicating that both domains are essential for protein function, structure formation, or localization.

### Analysis of the cellular localization of partially deleted Mms6

**Figure 2:**
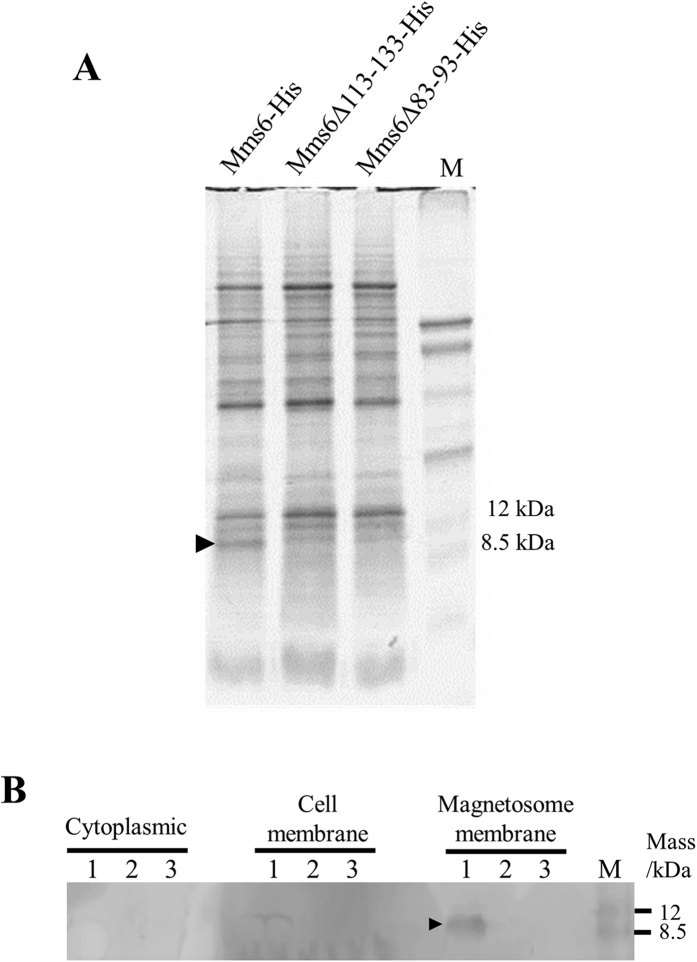
Analysis of the expression of His-tag-fused Mms6, Mms6Δ113*–*133, and Mms6Δ83*–*93 by SDS-PAGE (**A**). Proteins were purified from magnetosome membrane, and approximately 40 μg was loaded in each lane. M: Rainbow marker (low range). The black arrowhead indicates the putative Mms6-His protein. Expression of His-tag fused Mms6, Mms6Δ113*–*133, and Mms6Δ83*–*93 was confirmed by western blotting (**B**). M: Rainbow marker (low range). Black arrowheads indicate the putative Mms6-His.

The N-terminal region of the Mms6 protein including the GL region was predicted to form a transmembrane domain^20^,^37^; therefore, this region was believed to be required for the localization of Mms6 to the magnetosome membrane. The morphological changes in magnetite crystal could be a result of the mislocalization of the Mms6 variant with a deleted GL repetitive region. In order to confirm the subcellular localization of Mms6 variants, His-tag-fused protein expression vectors (pRKmms6-His, pRKmms6Δ113*–*133-His, and pRKmms6Δ83*–*93-His) were transformed into the Δ*mms6* strain. Protein profiles of magnetosome membrane fraction extracted from the crystals synthesized in the transformants were analyzed by sodium dodecyl sulfate-polyacrylamide gel electrophoresis (SDS-PAGE) (Fig. 2A). The predicted sizes of His-tag fused Mms6, Mms6Δ113*–*133, and Mms6Δ83*–*93 are approximately 6.8, 4.4, and 5.9 kDa, respectively. A band corresponding to the size of Mms6-His was observed at approximately 9 kDa in the lane of Mms6-His in the magnetosome membrane fraction. However, intelligible bands corresponding to the size of Mms6Δ113*–*133 and Mms6Δ83*–*93 were not identified in all the fractions by SDS-PAGE. It is noted that weaker bands whose band intensities are approximately 10% of Mms6-His were also observed at 9 kDa in both Mms6Δ113*–*133-His and Mms6Δ83*–*93-His samples. However, the band sizes are different from their predicted sizes, and thus the 8-kDa bands are most likely to be other magnetosome proteins, such as Mms5. No other significant difference in the patterns of the other protein bands was observed among the three samples. The SDS-PAGE gel was then subjected to western blotting. The band of Mms6-His was observed in the protein fraction obtained from magnetosome membrane (Fig. 2B). However, western blot analysis showed no visible amounts of Mms6Δ113*–*133-His and Mms6Δ83*–*93-His in the magnetosome membrane (Fig. 2B). This result suggests that the partial deletions in the Mms6 protein affect their expression or localization to magnetosomes or their protein structures, which also leads to protein mislocalization in the cell.

### Site-directed mutagenesis of acidic amino acids in Mms6

**Figure 3:**
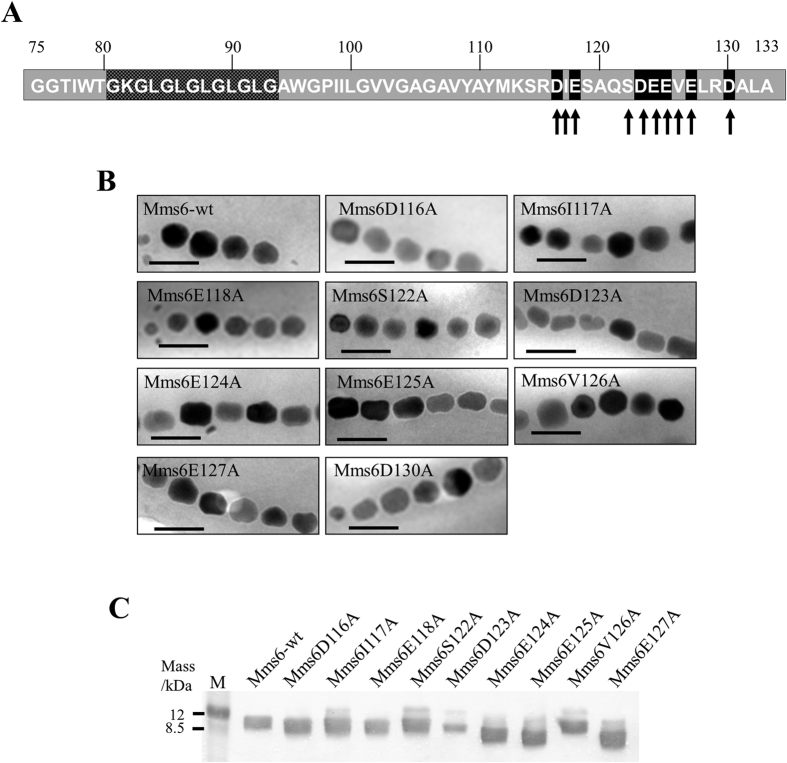
Characterization of Δ*mms6* strains harboring plasmids expressing a single amino acid substituted Mms6 protein. (**A**) Location of amino acid substitutions in the Mms6 protein. The acidic or non-acidic amino acid residues substituted by alanine are indicated by black and gray arrows, respectively. (**B**) Transmission electron micrographs of magnetite crystals in the Δ*mms6* strains expressing wild-type Mms6 or amino acid mutant derivatives. Scale bar, 100 nm. (**C**) Western blotting analysis of His-tag fused Mms6 variants substituted with single amino acid residues. His-tag fused protein expression vectors were transformed into the Δ*mms6* strain. Proteins were purified from magnetosome membrane, and approximately 40 μg was loaded in each lane. M: Rainbow marker (low range).

The deletion of large regions in the Mms6 protein sequence was theorized to be responsible for the lack of proteins in the cell fractions. Specifically, the N-terminal hydrophobic region of Mms6 has been previously implicated in protein localization and/or self-assembly^31^,^38^. The C-terminal region was theorized to be involved in the interaction of proteins with minerals such as an iron ion, iron hydroxide, and magnetite^20^,^32^. The influence of the acidic amino acid residues on the magnetite crystal formation was verified by individually replacing 7 acidic amino acids in the C-terminal region with alanine residues (Fig. 3A). Three non-acidic amino acid residues flanking the acidic residues were also substituted with alanine (Fig. 3A) for comparison. The Δ*mms6* strain harboring pRKmms6D123A, pRKmms6E124A, and pRKmms6E125A synthesized rod-shaped crystals, similar to those synthesized by the Δ*mms6* strain (Fig. 3B). In contrast, crystals produced by the Δ*mms6* strain harboring pRKmms6D116A, pRKmms6I117A, pRKmms6E118A, pRKmms6S122A, pRKmms6V126A, pRKmms6E127A, and pRKmms6D130A were spherical, similar to those produced by the wild-type strain and the Δ*mms6* strain harboring pRKmms6-wt (Fig. 3B).

The shape factors of the crystals produced by the Δ*mms6* strain harboring pRKmms6D123A, pRKmms6E124A, and pRKmms6E125A were in the range 0.65–0.66, which are distinct from those of the crystals produced by the Δ*mms6* strains harboring pRKmms6D116A, pRKmms6E118A, pRKmms6E127A, and pRKmms6D130A (shape factor: 0.78–0.87) (Mann-Whitney *p*-value < 0.05) (Table 2 and Supplementary Table S4). Scatterplot for the results of the principal component analysis showed that the crystals in the Δ*mms6* strain harboring pRKmms6D123A were more elliptical in comparison with the Δ*mms6* strains harboring pRKmms6S122A or pRKmms6E118A (Supplementary Figure 2). In addition, the substitution of non-acidic residues in the Δ*mms6* strains harboring pRKmms6I117A, pRKmms6S122A, and pRKmms6V126A did not affect the formation of spherical crystals (shape factor: 0.82–0.87) (Table 2), suggesting that these are non-functional residues.

To confirm the localization of Mms6 variants substituted with single amino acid residues, magnetosome membrane fractions from the transformants harboring His-tag-fused protein expression vectors were purified and analyzed. Western blot analysis revealed that all the variants of single amino acid substituted Mms6 (Mms6D116A, Mms6I117A, Mms6E118A, Mms6S122A, Mms6D123A, Mms6E124A, Mms6E125A, Mms6V126A, Mms6E127A) were expressed and localized in the magnetosome membrane (Fig. 3C). These results clearly indicated that the single amino acid substitutions at D123, E124, and E125 were responsible for the morphological change of the magnetite crystals. Thus, direct involvement of D123, E124, and E125 amino acid residues in the magnetite crystal formation is suggested. The bands for Mms6E124A, Mms6E125A, and Mms6E127A appeared at a position corresponding to a molecular weight lower than that of wild-type Mms6, probably due to the influence of the amino acid substitutions (Fig. 3C). Relatively weak expression of Mms6E123A may be due to the mislocalization or impairment of the direct interaction between Mms6 and the magnetite crystal surface.

## Discussion

The magnetite biomineralization process in magnetotactic bacteria is comprised of multiple steps, including vesicle formation^27^,^39^, assembly of the vesicle into a chain structure along the filament protein^40^,^41^, sorting of protein^42^, iron transport^43^, redox control in the vesicles^44^,^45^,^46^, and crystal formation^29^,^30^,^47^. Mms6 protein is a key protein for the crystal formation, and most probably localizes to the magnetosome membrane before or during the crystal nucleation^20^,^29^,^31^. Mms6 protein with eliminated GL region, which is predicted to be a transmembrane region, was abolished from the magnetosome membrane. The subcellular localization analysis indicated that elimination of this putative transmembrane region resulted in the change in localization of the Mms6 protein. Alternatively, the Mms6 variants are thought to be digested by endogenous proteases prior to the localization of the protein to the proper position in the magnetosome membrane^48^ because the His-tag fused proteins were also absent in the cytoplasm and cell membrane fractions. Moreover, elimination of the C-terminal region influenced the localization of proteins onto the surface of magnetite crystals. This suggests that direct association of Mms6 to the crystal surface is also important for the proper localization of this protein.

The interactions between the protein and crystal surface are believed to be important for biomineral formation^49^. The interaction of biomineral protein with the specific crystal face inhibits the crystal growth and is known to control the biomineral morphology^8^. Investigations into the possible mechanisms of interaction between several biomineral proteins and crystal surfaces have been proposed. Osteopontin is specifically adsorbed onto {100} faces of calcium oxalate monohydrate as a result of electrostatic interactions with its acidic amino acid and phosphate residues^8^. Statherin has a hydroxyapatite recognition region comprising of 15 amino acids in the N-terminal region, which causes the formation of a helical structure^50^. Four amino acid residues in this helical structure are positioned to associate with the hydroxyapatite surface^51^. Analysis of the X-ray crystal structure of osteocalcin revealed that the location of 5 acidic amino acids in the α-helix of this protein corresponds to the placement of calcium ions on the surface of hydroxyapatite^10^. Owing to this structural feature, osteocalcin can recognize a specific crystal surface of hydroxyapatite. These studies attributed the interactions between protein and mineral surfaces to negatively charged residues matching the crystal lattice.

The key residues crucial for the function of Mms6 protein were identified by establishing expression vectors for single amino acid-substituted Mms6 and transforming the same into the Δ*mms6* strain. The substitution of 3 consecutive acidic amino acids, Asp123, Glu124, and Glu125, impaired the function of Mms6 protein as a morphological regulator of magnetite crystal in magnetotactic bacteria. *In vitro* analyses of mutated Mms6 suggested that the C-terminal region affects the iron-binding ability and stability of the self-assembled protein structure^34^. The self-assembled C-terminal domains of Mms6 may form a macromolecular interface with appropriate spacing among the acidic amino acid residues, enabling its interaction with the specific crystal surface of magnetite^41^. The deletion of the *mms6* gene in *M. magneticum* AMB-1 resulted in the expression of {110} and high-index faces in the crystals^29^,^30^. As these faces are uncommon in the crystals from the wild-type strain, Mms6 protein was suggested to be involved in facilitating the formation of {110} face^30^. These suggested that the 3 acidic amino acid residues may directly associate with the {110} crystal face. The crystals synthesized by the Δ*mms6* strain were smaller than those produced by the wild-type strain, indicating that Mms6 protein is involved in crystal growth^29^,^30^. A previous study analyzing the crystallization of hydroxyapatite on a collagen fibril using poly-l-aspartic acid showed that poly-l-aspartic acid induced crystallization; this attributed the local super-saturation of calcium ions surrounding the fibril to the interactions between poly-l-aspartic acid and collagen^52^. Similarly, the amino acid residues of Mms6 protein may also be involved in the development of local super-saturation of iron ions that facilitate the growth of a specific crystal face.

In conclusion, both the C-terminal acidic amino acid region and N-terminal hydrophobic region including GL repetitive sequence are crucial for protein conformation and localization on the surface of magnetite crystals. The continuous acidic amino acids, Asp123, Glu124, and Glu125, in the C-terminal region, are key residues affecting the function of Mms6. Consequently, these three consecutive acidic amino acids play an essential role in the growth of crystals and morphological regulation of bacterial magnetite in *M. magneticum* AMB-1. Although other residues, such as basic amino acids, are important for biomineral-protein interaction^51^, our results correspond to the results of the *in vitro* and structural analyses of biomineral-protein interactions caused by acidic amino acids. Because acidic proteins commonly exist in biominerals, further experimental analyses may expand our understanding of *in vivo* biomineralization.

## Methods

### Strains and growth conditions

The strains, plasmids, and primers used in this study are summarized in Table S1–S3. *Escherichia coli* Top10 (Life Technologies, Carlsbad, CA, USA) and *E. coli* HST04 *dam*^−^/*dcm*^−^ (TaKaRa Bio Inc., Otsu, Japan) were used for gene cloning. *E. coli* cells were cultured in LB medium at 37 °C after addition of the appropriate antibiotics. The *E. coli* strain S17-1 was used as the donor in conjugation experiments and cultivated as previously described^23^. *M. magneticum* AMB-1 (ATCC700264) was anaerobically grown in a 30-mL vial. Colonies of *M. magneticum* AMB-1 were obtained on magnetic spirillum growth medium (MSGM) incubated micro-aerobically at 28 °C.

### Establishment of partial gene deletion mutants of *mms6*

The partially deleted Mms6 proteins constructed in this study are listed in Fig. 1A. The *mms6* sequence was obtained from the National Center for Biotechnology Information (NCBI) (YP_420319.1). In-frame partial deletions were introduced into *mms6* using plasmid vectors that contain DNA amplified from the region 500-bp upstream and downstream of the desired fragments, using primers listed in Table S3. These amplified fragments were digested and ligated to pK19mobsacB^53^, generating the plasmid vectors pK19M6d-1 and pK19M6d-2. The gentamicin resistance gene was also amplified, digested, and ligated into the *Xba*I site of the plasmids, generating plasmids pK19M6dGm^r^-1 and pK19M6dGm^r^-2. A contiguous sequence of pK19mobsacB, gentamicin resistance gene, and downstream of *mms6* (pK19Gm^r^d) were amplified using the primers, pK19Gmdown-F and pK19Gmdown-R. A 500-bp fragment upstream of the desired deletion was amplified using the appropriate primers, and ligated to the pK19Gm^r^d, generating pK19M6dGm^r^-3 and pK19M6dGm^r^-4. The N-terminal GL region was deleted using a 500-bp fragment upstream of the *mms6* stop codon synthesized by TaKaRa Bio Inc. that lacked the sequence coding for the GL region. This fragment was also ligated to the pK19Gm^r^d, generating pK19M6dGm^r^-5. Similarly, the internal region between the N-terminal GL region and the C-terminal acidic region was deleted by synthesizing a 500-bp fragment upstream of the *mms6* stop codon, which deleted the sequence coding for the internal region (TaKaRa Bio Inc.). This fragment was ligated to pK19Gm^r^d, generating pK19M6dGm^r^-6. The acidic amino acids in Mms6 were substituted with lysine residues by synthesizing a 500-bp fragment upstream of the *mms6* stop codon replacing the glutamic and aspartic acid residues with lysine (TaKaRa Bio Inc.). This fragment was also ligated to the pK19Gm^r^d, generating pK19M6dGm^r^-7.

The constructed plasmids were conjugated into *M. magneticum* AMB-1, as described in our previous report^23^. The obtained colonies were analyzed by PCR for the presence of the gentamicin resistance gene and the absence of the target gene. The presence of *mms5, mms7,* and *mms13* genes was also confirmed by PCR. The correct replacement of the *mms6* gene with the mutant genes and gentamicin gene was confirmed by DNA sequencing.

### Complementation of *mms6* with partial deletions in the Δ*mms6* strain

The Δ*mms6* was genetically complemented by preparing a pRK415^54^ -based plasmid harboring the promoter region of the *mms6* gene, and a partially deleted or partially substituted *mms6* gene (Table S2). The DNA fragments encoding Mms6, the truncated form of the Mms6 protein (Mms6Δ113*–*133), and the internal deletion form of Mms6 (Mms6Δ83*–*93) were amplified using the primers listed in Table S3. In addition, the DNA fragment encoding the Mms6 protein with substituted acidic amino acids (Mms6K) was also amplified using the primers listed in Table S3. Plasmids expressing a His-tag-fused Mms6 (Mms6-His) and a partially deleted Mms6 (Mms6Δ113*–*133-His and Mms6Δ83*–*93-His) were also established. Additionally, single amino acid substituted Mms6 protein (Mms6D116A, Mms6E118A, Mms6D123A, Mms6E124A, Mms6E125A, Mms6E127A, and Mms6 D130A)-expressing plasmids were constructed using QuickChange II XL site-directed mutagenesis kits (Agilent Technologies, Inc., Santa Clara, CA, USA). The plasmids were electroporated into Δ*mms6* as described in a previous study^24^.

### TEM analysis of magnetite crystals

Low magnification TEM analysis was performed using a conventional TEM (JEM1200-EX; JEOL Ltd., Tokyo, Japan) at 80 or 100 kV. Major and minor axes of magnetite crystal are defined as the maximum diameter of the particle and the orthogonal maximum diameter to the major axis respectively (Supplementary Figure 1). At least 105 crystals produced by each strain were analyzed, and the crystal size (average major axis and minor axis) and shape factor (ratio minor axis/major axis) was evaluated. The cells were randomly selected and only crystals with sizes ≥ 5 nm were measured in this study.

### Protein localization analysis by western blotting

Magnetite crystals were extracted from 10 L of culture (Δ*mms6*-pRKmms6-His, Δ*mms6*-pRKmms6Δ113-133-His, and Δ*mms6*-pRKmms6Δ83*–*93-His) and washed 5 times with HEPES (pH 7.0). The other cell fractions were also prepared as described in a previous report^22^. Magnetosome proteins were isolated by treating magnetite crystals with 1% (w/v) SDS in a 100 °C water bath for 30 min. Protein concentrations in the solution were measured using a standard protein assay kit (Bio-Rad, Hercules, CA, USA), using bovine serum albumin (BSA) as the standard and adjusted to the required concentration prior to electrophoresis. The localization of the His-tag fused Mms6 (Mms6-His) and partially deleted Mms6 (Mms6Δ113*–*133-His and Mms6Δ83*–*93-His) proteins were analyzed by SDS-PAGE; the separated proteins were then transferred to polyvinylidene difluoride (PVDF) membranes and probed using anti-His antibodies (Sigma–Aldrich, St. Louis, MO, USA). The SDS-polyacrylamide gel was stained with Bio Safe-Coomassie G-250 (Bio-Rad).

## Additional Information

**How to cite this article**: Yamagishi, A. *et al*. Core Amino Acid Residues in the Morphology-Regulating Protein, Mms6, for Intracellular Magnetite Biomineralization. *Sci. Rep.* **6**, 35670; doi: 10.1038/srep35670 (2016).

## Supplementary Material

Supplementary Information

## Figures and Tables

**Table 1:** Characteristics of magnetite crystals produced by recombinants carrying partially deleted Mms6 proteins and the Δ*mms6* strain, complemented with a partially deleted Mms6.

Strain	Plasmid	Crystal size (nm)	Major axis (nm)	Minor axis (nm)	Shape factor
Wild-type	—	39.8 ± 11.5	41.4 ± 12.5	38.1 ± 11.8	0.88 ± 0.10
*mms6*Δ*123–133*	—	31.8 ± 15.2	36.0 ± 16.6	27.6 ± 14.1	0.76 ± 0.12
*mms6*Δ*113–133*	—	30.7 ± 9.4	34.9 ± 10.6	26.5 ± 8.7	0.76 ± 0.14
*mms6K*	—	30.8 ± 9.2	35.6 ± 10.4	25.9 ± 8.6	0.73 ± 0.11
*mms6*Δ*83–93*	—	30.3 ± 13.1	35.5 ± 14.5	25.1 ± 12.1	0.70 ± 0.13
*mms6*Δ*94–112*	—	29.2 ± 12.6	35.3 ± 14.3	23.0 ± 11.4	0.64 ± 0.13
*mms6*Δ*94–133*	—	33.3 ± 13.2	40.3 ± 15.8	26.2 ± 11.2	0.66 ± 0.12
*mms6*Δ*83–133*	—	27.3 ± 13.6	33.3 ± 15.5	21.2 ± 12.3	0.63 ± 0.14
Δ*mms6*	—	35.5 ± 12.5	42.3 ± 14.6	27.9 ± 11.1	0.64 ± 0.12
Δ*mms6*	pRKmms6-wt	41.4 ± 14.0	43.9 ± 14.2	38.8 ± 14.0	0.88 ± 0.09
Δ*mms6*	pRKmms6Δ113–133	32.1 ± 12.4	38.3 ± 14.1	25.9 ± 11.2	0.67 ± 0.11
Δ*mms6*	pRKmms6K	30.9 ± 13.2	37.0 ± 15.0	24.8 ± 11.9	0.66 ± 0.12
Δ*mms6*	pRKmms6Δ83–93	31.3 ± 12.4	38.0 ± 14.5	24.6 ± 10.6	0.65 ± 0.11

Data represents the mean ± standard deviation. Crystal size is the average of major and minor axes. Shape factor is calculated as minor axis divided by major axis (minor/major axis). At least 175 crystals were evaluated for each strain. - : no plasmid was transformed.

**Table 2:** Characteristics of magnetite crystals expressed in transformants expressing acidic amino acid mutant derivatives.

Strain	Plasmid	Crystal size (nm)	Major axis (nm)	Minor axis (nm)	Shape factor
Δ*mms6*	pRKmms6-wt	41.4 ± 14.0	43.9 ± 14.2	38.8 ± 14.0	0.88 ± 0.09
Δ*mms6*	pRKmms6I117A	45.0 ± 13.3	44.5 ± 13.3	37.3 ± 12.0	0.83 ± 0.11
Δ*mms6*	pRKmms6D116A	40.2 ± 11.7	44.1 ± 12.8	36.3 ± 11.1	0.83 ± 0.11
Δ*mms6*	pRKmms6E118A	38.3 ± 13.0	40.9 ± 13.7	35.8 ± 12.5	0.87 ± 0.08
Δ*mms6*	pRKmms6S122A	39.5 ± 12.5	43.1 ± 13.1	35.8 ± 12.4	0.82 ± 0.12
Δ*mms6*	pRKmms6D123A	32.5 ± 12.4	39.6 ± 14.9	25.4 ± 10.7	0.65 ± 0.13
Δ*mms6*	pRKmms6E124A	37.4 ± 13.5	44.6 ± 14.9	30.1 ± 12.8	0.66 ± 0.13
Δ*mms6*	pRKmms6E125A	34.1 ± 12.7	40.7 ± 14.2	27.5 ± 11.7	0.66 ± 0.12
Δ*mms6*	pRKmms6V126A	40.9 ± 12.9	43.7 ± 13.6	38.1 ± 12.6	0.87 ± 0.09
Δ*mms6*	pRKmms6E127A	39.9 ± 13.0	44.6 ± 14.2	35.1 ± 12.1	0.78 ± 0.09
Δ*mms6*	pRKmms6D130A	38.9 ± 13.8	43.5 ± 15.1	34.3 ± 12.9	0.79 ± 0.12

Data represents the mean ± standard deviation. Crystal size is the average of major and minor axes. Shape factor is calculated as minor axis divided by major axis (minor/major axis). At least 105 crystals were evaluated for each strain.
