# Supplementary material for: Core Amino Acid Residues in the Morphology-Regulating Protein, Mms6, for Intracellular Magnetite Biomineralization
Source: Sci Rep. 2016 Oct 19;6:35670. doi: 10.1038/srep35670 (PMC5069546; doi:10.1038/srep35670)
Supplement: Supplementary Information [file srep35670-s1.pdf]

Supporting Information

Core Amino Acid Residues in the Morphology-Regulating Protein, Mms6, for Intracellular Magnetite Biomineralization

Ayana Yamagishi, Kaori Narumiya, Masayoshi Tanaka, Tadashi Matsunaga, Atsushi Arakaki*

Supplementary Table S1. Strains used in this study

Strains	Description	Source or reference
E. coli Top10	General cloning	Life Technologies
E. coli S17-1	Mobilizing strain	Simon et al. (1983)
E. coli HST04 *dam*⁻/*dcm*⁻ Competent Cells	Cloning of pK19M6d-1 and pK19M6d-2	TaKaRa
M. magneticum AMB-1	Wild-type strain	ATCC700264
Δ*mms6*	*mms6* deleted mutant strain	Tanaka et al. (2011)
mms6Δ123-133	Mms6 mutant with partial deletion of amino acid residue 49 through 59	This study
mms6Δ113-133	Mms6 mutant with partial deletion of amino acid residue 39 through 59	This study
mms6Δ94-133	Mms6 mutant with partial deletion of amino acid residue 20 through 59	This study
mms6Δ83-133	Mms6 mutant with partial deletion of amino acid residue 9 through 59	This study
mms6K	Mutant designed by replacing the acidic amino acids of Mms6 protein from amino acid residues 39 to 59 with lysine residues.	This study
mms6Δ83-93	Mms6 mutant with partial deletion of amino acid residue 9 through 19	This study
mms6Δ94-112	Mms6 mutant with partial deletion of amino acid residue 20 through 38	This study
Δ*mms6*-pRKmms6-wt	Δ*mms6* strain carrying vector pRKmms6-wt	This study
Δ*mms6*-pRKmms6Δ113-133	Δ*mms6* strain carrying vector pRKmms6Δ113-133	This study
Δ*mms6*-pRKmms6Δ83-93	Δ*mms6* strain carrying vector pRKmms6Δ83-93	This study
Δ*mms6*-pRKmms6K	Δ*mms6* strain carrying vector pRKmms6K	This study
Δ*mms6*- pRKmms6-wt-His	Δ*mms6* strain carrying vector pRKmms6-wt-His	This study
Δ*mms6*-pRKmms6Δ113-133-His	Δ*mms6* strain carrying vector pRKmms6Δ113-133-His	This study
Δ*mms6*-pRKmms6Δ83-93-His	Δ*mms6* strain carrying vector pRKmms6Δ83-93-His	This study
Δ*mms6*-pRKmms6D116A	Δ*mms6* strain carrying vector pRKmms6D116A	This study
Δ*mms6*-pRKmms6E118A	Δ*mms6* strain carrying vector pRKmms6E118A	This study
Δ*mms6*-pRKmms6D123A	Δ*mms6* strain carrying vector pRKmms6D123A	This study
Δ*mms6*-pRKmms6E124A	Δ*mms6* strain carrying vector pRKmms6E124A	This study
Δ*mms6*-pRKmms6E125A	Δ*mms6* strain carrying vector pRKmms6E125A	This study
Δ*mms6*-pRKmms6E127A	Δ*mms6* strain carrying vector pRKmms6E127A	This study
Δ*mms6*-pRKmms6D130A	Δ*mms6* strain carrying vector pRKmms6D130A	This study

Supplementary Table S2. Plasmids used in this study

Plasmids	Description	Source or reference
pK19mobsacB	*Km*^{r}, *sacB*, *lacZ*	Schafer et al. (1994)
pK19M6dGm^r-1	pK19mobsacB with upstream1 and downstream of *mms6* and *Gm*^{r}	This study
pK19M6dGm^r-2	pK19mobsacB with upstream2 and downstream of *mms6* and *Gm*^{r}	This study
pK19M6dGm^r-3	pK19mobsacB with upstream3 and downstream of *mms6* and *Gm*^{r}	This study
pK19M6dGm^r-4	pK19mobsacB with upstream4 and downstream of *mms6* and *Gm*^{r}	This study
pK19M6dGm^r-5	pK19mobsacB with upstream5 and downstream of *mms6* and *Gm*^{r}	This study
pK19M6dGm^r-6	pK19mobsacB with upstream6 and downstream of *mms6* and *Gm*^{r}	This study
pK19M6dGm^r-7	pK19mobsacB with upstream7 and downstream of *mms6* and *Gm*^{r}	This study
SYN4030 2-3	pMD19T with upstream5	TakaRa
SYN4030 3-4	pMD19T with upstream6	TakaRa
SYN4444	pMD19T with upstream7	TakaRa
pRK415	Mobilizable broad-host-range vector; origin of replication (oriV), and origin of transfer (oriT) of RK2; Tcr	Keen et al. (1988)
pRKmms6-wt	pRK415 with P_{mms6} and *mms6* gene	This study
pRKmms6Δ113-133	pRK415 with P_{mms6} and partial deleted *mms6* gene cording Mms6Δ113-133	This study
pRKmms6Δ83-93	pRK415 with P_{mms6} and partial deleted *mms6* gene cording Mms6Δ83-93	This study
pRKmms6K	pRK415 with P_{mms6} and partial deleted *mms6* gene cording Mms6K	This study
pRKmms6His-wt	pRK415 with P_{mms6} and His-tag fused *mms6* gene	This study
pRKmms6Δ113-133-His	pRK415 with P_{mms6} and His-tag fused partial deleted *mms6* gene cording Mms6Δ113-133	This study
pRKmms6Δ83-93-His	pRK415 with P_{mms6} and His-tag fused partial deleted *mms6* gene cording Mms6Δ83-93	This study
pRKmms6D116A	pRK415 with P_{mms6} and 1 amino acid substituted *mms6* gene cording Mms6D116A	This study
pRKmms6I117A	pRK415 with P_{mms6} and 1 amino acid substituted *mms6* gene cording Mms6I117A	This study
pRKmms6E118A	pRK415 with P_{mms6} and 1 amino acid substituted *mms6* gene cording Mms6E118A	This study
pRKmms6S122A	pRK415 with P_{mms6} and 1 amino acid substituted *mms6* gene cording Mms6S122A	This study
pRKmms6D123A	pRK415 with P_{mms6} and 1 amino acid substituted *mms6* gene cording Mms6D123A	This study
pRKmms6E124A	pRK415 with P_{mms6} and 1 amino acid substituted *mms6* gene cording Mms6E124A	This study
pRKmms6E125A	pRK415 with P_{mms6} and 1 amino acid substituted *mms6* gene cording Mms6E125A	This study
pRKmms6V126A	pRK415 with P_{mms6} and 1 amino acid substituted *mms6* gene cording Mms6V126A	This study
pRKmms6E127A	pRK415 with P_{mms6} and 1 amino acid substituted *mms6* gene cording Mms6E127A	This study
pRKmms6D130A	pRK415 with P_{mms6} and 1 amino acid substituted *mms6* gene cording Mms6D130A	This study
pRKmms6D116A-His	pRK415 with P_{mms6} and gene cording His-tag-fused Mms6D116A	This study
pRKmms6I117A-His	pRK415 with P_{mms6} and gene cording His-tag-fused Mms6I117A	This study
pRKmms6E118A-His	pRK415 with P_{mms6} and gene cording His-tag-fused Mms6E118A	This study

pRKmms6S122A-His	pRK415 with P_{mms6} and gene cording His-tag-fused Mms6S122A	This study
pRKmms6D123A-His	pRK415 with P_{mms6} and gene cording His-tag-fused Mms6D123A	This study
pRKmms6E124A-His	pRK415 with P_{mms6} and gene cording His-tag-fused Mms6E124A	This study
pRKmms6E125A-His	pRK415 with P_{mms6} and gene cording His-tag-fused Mms6E125A	This study
pRKmms6V126A-His	pRK415 with P_{mms6} and gene cording His-tag-fused Mms6V126A	This study
pRKmms6E127A-His	pRK415 with P_{mms6} and gene cording His-tag-fused Mms6E127A	This study

Supplementary Table S3. Primers used in this study

Primers	Description	Source or reference
mms6up1-F	5′-TCGCCGGAATTCCAGCAATGATCTCATGTTATTGATG-3′	This study
mms6up1-R	5′-GCTTCCTCTAGATCACATATACGCGTAAACCGCCC-3′	This study
mms6up2-F	5′-GCTTTCGAATTCAGTCTGAAGGGGGGCTGGCG-3′	This study
mms6up2-R	5′-GATTCGTCTAGATCAGCTCTGCGCCGATTCGATATC-3′	This study
mms6up3-F	5′-ACGGCAATGCATAATAGTTGGTCGCAACTT-3′	This study
mms6up3-R	5′-AGATACAGTACTTCAGCCCAGACCGAGACC-3′	This study
mms6up4-F	5′-ATTATTATGCATCTAATTCTCTTTTGCTCTTCTGAG-3′	This study
mms6up4-R	5′-ATTATTAGTACTTCACTTACCGGTCCAGATGGTTCC-3′	This study
pK19Gmdown-F	5′-GGGAGGAAGTACTGTCGATGTTTGATGTTATGG-3′	This study
pK19Gmdown-R	5′-AACAACATGCATACTGGCCGTCGTTTTACAAC-3′	This study
Gm^r-F	5′- TTTCCCTCTAGAGTCGATGTTTGATGTTATG-3′	This study
Gm^r-R	5′-ATTATATCTAGATTAGGTGGCGGTACTTGGGT-3′	This study
pk19100-F	5′- GCTATTACGCCAGCTGGCGAAAG-3′	This study
mms6-F	5′-GCGCTACTTGATTGTGAGGAGAACC-3′	This study
mms6-R	5′-AAGCACAAGACGCTGAGATAGGAC-3′	This study
mms5-F	5′-GCGGTCATTTCGTCCTCCT-3′	Arakaki et al. (2014)
mms5-R	5′-GGCAGCACAGATGGCAACA-3′	Arakaki et al. (2014)
mms7-F	5′-GGCATTGACAATGTCGCCGATGGTC-3′	Arakaki et al. (2014)
mms7-R	5′-CCAAGCCGACCAGGATTGAATACGTC-3′	Arakaki et al. (2014)
mms13-F	5′-GATCACCAAGGCTGGGGACCG-3′	This study
mms13-R	5′-GCATTCTCTGCTTCGTCCCGCTG-3′	This study
mms6-F	5′-ATTAAACTGCAGGTGCCAGCTCAGATCG-3′	This study
mms6_1-R	5′-GCTTCCAAGCTTTCACATATACGCGTAAACCG-3′	This study
mms6_2-R	5′-TTTTTTAAGCTTTCAGGCCAGCGCGTCG-3′	This study
mms6_3-R	5′-TTTTTTAAGCTTTCAGGCCAGCGCTTTGC-3′	This study
Pmms6-F	5′-GCTTTTGAATTCTTCTCTTTTGCTCTTCTGAGAGA-3′	This study
Pmms6-R	5′-TTTTTTCTGCAGTCTCCTCACAATCAAGTAGCG-3′	This study
mms6His_1-R	5′-TACGCCAAGCTTTCAGTGGTGATGGTGATGATGCATATACGCG TAAAC-3′	This study
mms6His_2-R	5′-TTTTTTAAGCTTTCAGTGGTGATGGTGATGATGGGCCAGCGCGT C-3′	This study
pRKd100-R	5′-GGCTTTACACTTTATGCTTCCGGCTCGTATGTTG-3′	This study
D116-F	5′-GAAGAGCCGTGCAATCGAATCGGCG-3′	This study
D116-R	5′-CCGATTCGATTGCACGGCTCTTCATATACG-3′	This study

I117-F	5′-GAGCCGTGATGCAGAATCGGCGCAG-3′	This study
I117-R	5′-GCGCCGATTCTGCATCACGGCTCTTC-3′	This study
E118-F	5′-GAAGAGCCGTGATATCGCATCGGCGCAGAG-3′	This study
E118-R	5′-GTCGCTCTGCGCCGATGCGATATCACGG-3′	This study
S122-F	5′-GAATCGGCGCAGGCAGACGAGGAAGTC-3′	This study
S122-R	5′-CCTCGTCTGCCTGCGCCGATTCGATATCAC-3′	This study
D123-F	5′-GAATCGGCGCAGAGCGCAGAGGAAGTCG-3′	This study
D123-R	5′-CAGTTCGACTTCCTCTGCGCTCTGCGCC-3′	This study
E124-F	5′-GCGCAGAGCGACGCAGAAGTCGAACTGCG-3′	This study
E124-R	5′-CAGTTCGACTTCTGCGTCGCTCTGCGCCGATTC-3′	This study
E125-F	5′-GAGCGACGAGGCAGTCGAACTGCGCGAC-3′	This study
E125-R	5′-GCAGTTCGACTGCCTCGTCGCTCTGCG-3′	This study
V126-F	5′-GCGACGAGGAAGCAGAACTGCGCGAC-3′	This study
V126-R	5′-CGCGCAGTTCTGCTTCCTCGTCGCTC-3′	This study
E127-F	5′-CGAGGAAGTCGCACTGCGCGACGCGC-3′	This study
E127-R	5′-CGTCGCGCAGTGCGACTTCCTCGTCGCTC-3′	This study
D130-F	5′-GTCGAACTGCGCGCAGCGCTGGCCTG-3′	This study
D130-R	5′-CAGGCCAGCGCTGCGCGCAGTTCGAC-3′	This study
pRK415_sequence_F	5′-CTCACTCATTAGGCACCCCAGG-3′	This study
pRK415_sequence_R	5′-GGATGTGCTGCAAGGCGATTAAG-3′	This study

Supplementary Table S4. Statistical analysis of magnetite crystals produced by the recombinants and complemented strains

Strain	Plasmid	Major axis		Minor axis		Shape factor	
		Mean ± SD (nm)	P-value	Mean ± SD (nm)	P-value	Mean ± SD (nm)	P-value
mms6Δ113-133	-	34.9 ± 10.6	0.25	26.5 ± 8.7	0.73	0.76 ± 0.14	0.63
mms6Δ123-133	-	36.0 ± 16.6		27.6 ± 14.1		0.76 ± 0.12	
mms6Δ83-93	-	35.5 ± 14.5	$1.2x10^{-4}$*	25.1 ± 12.1	0.2	0.70 ± 0.13	$9.2x10^{-5}$*
mms6Δ94-133	-	40.3 ± 15.8		26.2 ± 11.2		0.66 ± 0.12	
mms6Δ83-93	-	32.0 ± 12.1	0.08	20.0 ± 8.9	$9.6x10^{-6}$*	0.63 ± 0.14	$1.4x10^{-9}$*
mms6Δ83-133	-	33.3 ± 15.5		21.2 ± 12.3		0.63 ± 0.14	
Δ*mms6*	pRKmms6-wt	43.9 ± 14.2	$7.7x10^{-6}$*	38.8 ± 14.0	$1.0x10^{-5}$*	0.88 ± 0.09	$7.2x10^{-4}$*
Δ*mms6*	pRKmms6Δ113-133	38.3 ± 14.1		25.9 ± 11.2		0.67 ± 0.11	
Δ*mms6*	pRKmms6-wt	43.9 ± 14.2	$1.1x10^{-6}$*	38.8 ± 14.0	$1.2x10^{-5}$*	0.88 ± 0.09	$7.3x10^{-4}$*
Δ*mms6*	pRKmms6K	37.0 ± 15.0		24.8 ± 11.9		0.66 ± 0.12	
Δ*mms6*	pRKmms6-wt	43.9 ± 14.2	$8.4x10^{-6}$*	38.8 ± 14.0	$2.3x10^{-5}$*	0.88 ± 0.09	$4.2x10^{-4}$*
Δ*mms6*	pRKmms6Δ83-93	38.0 ± 14.5		24.6 ± 10.6		0.65 ± 0.11	
Δ*mms6*	pRKmms6E127A	44.6 ± 14.2	$3.8x10^{-3}$*	35.1 ± 12.1	$1.4x10^{-9}$*	0.78 ± 0.09	$8.7x10^{-7}$*
Δ*mms6*	pRKmms6D123A	39.6 ± 14.9		25.4 ± 10.7		0.65 ± 0.13	
Δ*mms6*	pRKmms6E127A	44.6 ± 14.2	0.6	35.1 ± 12.1	$1.1x10^{-3}$*	0.78 ± 0.09	$1.1x10^{-15}$*
Δ*mms6*	pRKmms6E124A	44.6 ± 14.9		30.1 ± 12.8		0.66 ± 0.13	
Δ*mms6*	pRKmms6E127A	44.6 ± 14.2	0.01*	35.1 ± 12.1	$9.6x10^{-8}$*	0.78 ± 0.09	$2.0x10^{-5}$*
Δ*mms6*	pRKmms6E125A	40.7 ± 14.2		27.5 ± 11.7		0.66 ± 0.12	

Data represent the mean ± standard deviation. The shape factor was calculated as the minor axis divided by the major axis (minor/major axis). The Mann-Whitney test was used to acquire P-values. *$P < 0.05$

References

Simon R, P. U., Pühler A. A broad host range mobilization system for *in vivo* genetic engineering: transposon mutagenesis in Gram-negative bacteria. *Nat. Biotechnol*. 1, 784 - 791 (1983).

Keen, N. T., Tamaki, S., Kobayashi, D. & Trollinger, D. Improved broad-host-range plasmids for DNA cloning in gram-negative bacteria. *Gene* 70, 191-197 (1988).

Schäfer, A. et al. Small mobilizable multi-purpose cloning vectors derived from the Escherichia coli plasmids pK18 and pK19: selection of defined deletions in the chromosome of Corynebacterium glutamicum. *Gene* 145, 69-73 (1994).

Tanaka, M., Mazuyama, E., Arakaki, A. & Matsunaga, T. MMS6 protein regulates crystal morphology during nano-sized magnetite biomineralization *in vivo*. *J Biol Chem* 286, 6386-6392, doi:10.1074/jbc.M110.183434 (2011).

Arakaki, A., Yamagishi, A., Fukuyo, A., Tanaka, M. & Matsunaga, T. Co-ordinated functions of Mms proteins define the surface structure of cubo-octahedral magnetite crystals in magnetotactic bacteria. *Mol Microbiol* 93, 554-567, doi:10.1111/mmi.12683 (2014).

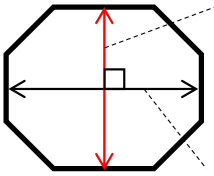

Supplementary Figure 1. Major and minor axes for statistical analysis of magnetite crystals. The major axis is defined as the maximum diameter of the magnetite crystals. The minor axis is the maximum diameter that crosses the major axis at a right angle.

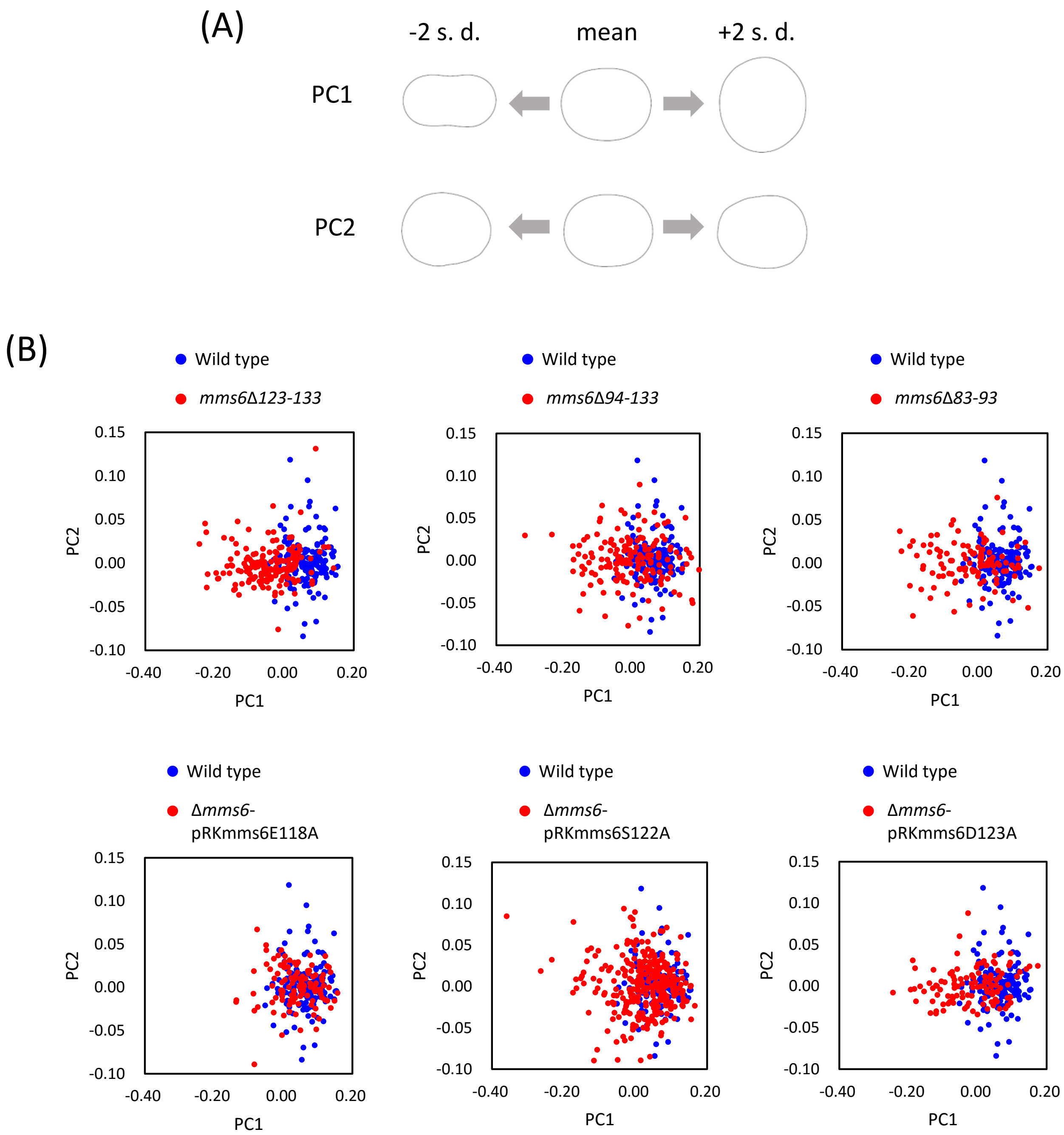

Supplementary Figure 2. Principal component analysis of elliptical Fourier descriptors for the shape of magnetite crystals. (A) Crystal shapes along the PC1 and PC2 axes. The patterns show a mean shape and shapes +2 and -2 standard deviation (SD) distant from the mean. (B) Scatterplot for the results of the principal component analysis of elliptic Fourier descriptors.
